# Learning Outputs for Peer Teachers in Undergraduate Medical Education

**DOI:** 10.1007/s40670-025-02365-0

**Published:** 2025-03-22

**Authors:** Idun Grimstad Skjærseth, Thomas Mildestvedt, Anna Bonnevier, Satya Pal Sharma, Monika Kvernenes

**Affiliations:** 1 Center for Medical Education & Department of Global Public Health and Primary Care, Faculty of Medicine, University of Bergen, Bergen, Norway; 2 Department of Global Public Health and Primary Care, Faculty of Medicine, University of Bergen, Bergen, Norway; 3 Unit for Teaching and Learning &, Department of Learning, Informatics, Management and Ethics, Karolinska Institutet, Stockholm, Sweden; 4 Department of Global Public Health and Primary Care, Faculty of Medicine, University of Bergen, Bergen, Norway; 5 Center for Medical Education &, Department of Clinical Medicine, Faculty of Medicine, University of Bergen, Bergen, Norway

**Keywords:** Peer teaching, Peer-assisted learning, Educator roles, Generic skills, Undergraduate medical education

## Abstract

Peer teaching is a commonly employed educational approach in medical education, providing valuable learning opportunities for both peer teachers and learners. While it is known to enhance subject knowledge for those in teaching roles, what broader learning outcomes can be achieved and the conditions enabling these outcomes remain less understood. Hence, our aim was to explore the learning outcomes experienced by peer teachers in undergraduate medical education, as well as to identify the prerequisites that facilitate these outcomes. Whereas most studies on peer teaching have focused on outcomes from evaluations of individual, newly implemented programs, this study examines peer teaching experiences from a variety of programs and contexts. To achieve a broader dataset, peer teachers (*n*=34) at four Scandinavian medical schools were recruited to participate in focus groups discussing their experience with peer teaching and what they had learned. Transcripts were analyzed using reflexive thematic analysis. The results demonstrate that peer teachers developed an understanding of the teacher’s role as facilitators of learning, recognized the limits of their own knowledge, learned to adapt their teaching to meet learners’ needs, and recognized the importance of psychological safety for learning. These learning outputs were facilitated by accessible and attractive teaching opportunities, a sense of responsibility and agency, sufficient preparation, and access to support during the teaching process. Overall, being a peer teacher helps medical students gain competencies that are useful in their future careers. Program leaders should design peer teaching opportunities that facilitate peer teachers’ learning.

**Supplementary Information:**

The online version contains supplementary material available at 10.1007/s40670-025-02365-0.

## Introduction

Peer teaching, where a student acts as a teacher for one or more of their fellow or more junior peers, is a commonly used educational strategy in medical education [1]. It is often implemented for the sake of convenience as it lessens teaching load for faculty [2]. However, other benefits have been described, both for peer learners and peer teachers: Learners have been shown to learn equally efficiently when being taught by peers or near-peers as by professionals [3, 4] and social and cognitive congruence contribute to favorable learning environments [1, 5–7]. As for the peer teachers, studies have demonstrated that they experience enhanced subject expertise [8–12]. Students who teach achieve higher exam scores [10], which can be explained partly by their interaction with the teaching content and partly by a selection bias [13], as high-achieving medical students seem to be attracted to the teaching role [14].

Furthermore, research suggests that peer teaching fosters a range of skills and abilities, including enhanced communication, greater confidence, deeper self-awareness, and contributes to professional identity formation [5, 8, 9, 11, 12, 14]. A systematic review by Tanveer et al. looked at learning outputs for peer teachers reported in the literature and found that peer teachers gain pedagogical knowledge and skills, personal outputs and generic skills as well as subject-specific learning outputs [15]. Avonts et al. demonstrated that learning outputs gained by peer teachers are long-lasting [9]. Additionally, peer teachers have been found to gain competencies related to the CanMEDS competency framework, demonstrating that being a peer teacher has a positive impact on the competencies of medical expert, collaborator, scholar, and professional [14]. However, knowledge gaps remain related to what factors contribute to peer teachers’ learning outputs. Apart from evidence that peer teacher training has been found to improve peer teachers’ understanding of educational principles and to boost confidence in teaching [16], we lack knowledge about what facilitates peer teachers’ learning.

Furthermore, most studies examining learning outcomes for peer teachers focus on individual peer teaching programs, predominantly measuring peer learners’ satisfaction or learning outcomes, while only secondarily addressing the learning benefits for peer teachers. They are often evaluative in nature, based on self-reports that are superficially analyzed and restricted to summarizing students’ perceptions of their own learning [15]. Hence, we do not know whether the reported learning is specific to the program under study, or to peer teaching experiences as such.

In this study, we explore peer teaching across a variety of contexts and programs with the aim of identifying generic learning outputs for peer teachers in undergraduate medical education. We also sought to identify the prerequisites that lead to these outputs, with the intent to develop knowledge that may help peer teaching programs foster educational benefits for peer teachers as well as peer learners.

## Materials and Methods

The study follows a qualitative research design using semi-structured focus group interviews to gather detailed insights into peer teachers’ experiences [17]. The data was analyzed from a constructivist orientation using reflexive thematic analysis [18].

### The Participants

Medical students with experience as peer teachers were recruited from three medical schools in Norway and one in Sweden. Each medical school has its own curriculum; however, all use an integrated curriculum model and predominantly teach basic sciences the first two years. Clinical training is introduced in the early years and is gradually intensified. Two of the programs include problem-based learning as a teaching method in parts of the program. All represent medium-to-large programs recruiting 160–230 students each year. In both countries, students apply to medical school based on high school diploma scores. They obtain their medical license upon completing a 6-year undergraduate medical program. The medical program in Sweden has recently changed the duration of the program from 5.5 to 6 years. The interviews were conducted with students partaking in the old 5.5-year program followed by a requirement to complete an 18–24-month clinical internship known as “Allmäntjänstgöring” (AT) and pass an associated exam before receiving a medical license.

For the purpose of our study, we included peer teaching programs that were scheduled as part of the formal undergraduate curriculum with a few selected students taking on the role as teachers. We are aware of differences in terminology between contexts, for example, teaching assistants and peer teachers are different positions in the USA, whereas they are often used interchangeably in Europe. In this study, we use the term “peer teacher” to mean students that have dedicated roles as teachers and are appointed by the institution. General collaborative learning activities where students take turns on teaching each other were excluded. We included students who taught groups of varying sizes and across different subjects to capture a broad spectrum of peer teaching experiences. We also allowed varied distances in academic years between teachers and learners [2].

Procedures for recruitment followed almost identical steps in Norway and Sweden respectively: In Norway, the first author (IGS) identified relevant peer teaching programs and contacted peer teachers in eligible programs via e-mail with an invitation to participate in the study. Starting with an initial group of recruited informants, we employed a snowball sampling method, encouraging informants to suggest other peer teachers from their respective peer teaching programs to volunteer for interviews. In Sweden, an invitation to participate in the study was sent through a learning management system to all students in courses identified as containing peer teaching activities, reaching approximately 300 medical students, urging peer teachers interested in participating in the study to contact the research team. Additional potential informants (peer teachers in physiology) were contacted directly through e-mail with invitation to participate. No compensation was offered; however, food and soft drinks were provided during the interviews with the Norwegian students.

### Data Collection

To guide the focus group discussions, a semi-structured interview guide was developed. The design of the guide was informed by relevant existing literature on peer teaching. We conducted two pilot interviews, one in Norway and one in Sweden. The purpose was to ensure that the questions were understandable to interviewees, that they produced relevant data, and to provide feedback to the interviewers on moderation technique. Participants were prompted to describe their experiences with peer teaching, to reflect on and discuss what they had learned from being peer teachers and what impact peer teaching had on their personal and professional development. We also enquired about their collaboration with faculty members, what support was given before and during teaching, and what motivated the students to take on peer teaching assignments. In addition, the Swedish focus groups included additional questions for a separate study focusing on peer teacher training. See the Appendix for details.

We did five focus groups in Norway and another five in Sweden. All the Norwegian interviews were executed in person by IGS and MK. The Swedish interviews were conducted through Zoom and executed by a senior medical student on the Swedish research team under the guidance of experienced qualitative researchers. In five of the groups, participants were grouped together with peer teachers from the same peer teaching program, while the other five groups were a mix of students with peer teaching experiences from various settings. The number of participants per group varied from two to six. The interviews lasted between 55 and 75 min and were carried out in the period between March 2022 and February 2023. Saturation was assumed to be reached when focus groups no longer brought new insight. Audio records of the focus group discussions were transcribed verbatim in Norwegian and Swedish which are mutually intelligible languages, before being subjected to analysis.

### Data Analysis

Transcripts were analyzed using reflexive thematic analysis adhering to the six steps described by Braun and Clarke [18]. NVivo 12 was used for data management. All authors took part in step 1 by carefully reading the interview transcripts and made notes of initial impressions. After sharing our impressions of the data and revisiting our research questions, IGS coded the transcripts using an inductive approach (step 2). To guide our analysis, we used a pragmatic definition of learning that did not tie us to any specific learning theory but allowed us to search broadly for indications of changes in behavior, strategies, perspectives, values, and understanding. The development of initial themes representing learning outputs was done by IGS and MK (step 3). During analysis, we iteratively revisited the data, initial codes, and suggested themes, in discussions with the author team (step 4). After multiple rounds of drafts and discussions, we agreed on the themes describing the learning outputs for peer teachers (step 5). Disagreements were settled through discussion within the author team.

A second analysis was done with specific focus on identifying prerequisites for learning, which in this context was understood as facilitative factors, relations, and processes that contributed significantly to learning. Following the initial coding by IGS, themes were developed iteratively in meetings where IGS and MK went back and forth between codes and suggested themes. Preliminary findings were discussed with the entire team of researchers before completion. Writing of the report was led by IGS in close collaboration with MK (step 6).

### Reflexivity

The research team represents various perspectives on peer teaching. IGS is a medical student who has previous experience as a peer teacher; AB and MK are both medical education researchers with theoretical interests in peer teaching, while TM and SS are medical doctors and general practitioners and part of the university teaching staff. Both have experience with including students as teachers in their courses.

### Ethical Considerations

Approvals for the project were collected in separate processes in Norway and Sweden, respectively. The project was registered in the University of Bergen’s data protection service ensuring compliance with all applicable data protection and privacy regulations (ID: R1607). We consulted the Health Research Ethical Board where the project was considered to be outside the scope of the Health Research Act. For the Swedish interviews, ethical approval was sought from the local Ethical Review Board (Dnr 2021–06443−01). The Ethical Review Board had no ethical objections to the project.

All interviews followed national and international ethical guidelines regarding research involving human subjects. Students received written information and gave written informed consent before taking part in the interviews. Participation was voluntary. All participants were pseudonymized in the process of transcribing the audiotaped interviews and were therefore not identifiable for the rest of the research team.

## Results

Our material included data from 34 peer teachers, of whom 18 (53%) were women. Their age varied from 21 to 32 years, and they were enrolled in the 2nd to the 6th year of medical school. Most had taught one or two different subjects as peer teachers, and some had been peer teachers for several cohorts of peers, thus gaining more experience. Additional details about the participants are summarized in Table 1.

**Table 1:** Information about study participants and peer teaching activities pr. focus group

Study site and number of participants in focus group	Level of study		Group size	Subjects taught	Teaching format	Training and/or preparations
	Peer teacher	Students				
University A, Norway (*N*=6)	6th year	6th year	3–8	Joint examination	Demonstration and practical training doing joint examinations	Subject-specific training (2×3 h lecture)
University A, Norway (*N*=3)	3rd–4th year	1st–2nd year	1–2 peer teachers per group of 20–50 students	Anatomy dissection	Lecture, demonstration and supervising students doing dissection	Practical—such as where to find equipment in the dissection room
University A, Norway (*N*=2)	3rd year	1st year	5–6	Patient communication	Discussion groups with formative feedback	Teacher training and subject-specific training (2×3 h focusing on teacher role, handling difficult situations, giving feedback)
University B, Norway (*N*=4)	3rd–5th year	1st–2nd year	8	A variety of subjects	Problem-based learning	Teacher training (4 focusing on PBL facilitation)
University C, Norway (*N*=3)	3rd–5th year	1st–6th year	5–10	Clinical examinations and procedures	Lectures, demonstration, discussions, feedback	No
University D, Sweden (*N*=3)	3rd–5th year and 3 graduates	1st year	4–12 4–12	Anatomy dissection Histology microscopy	Small group teaching	Teacher training (1–3 h focusing on what subjects or activities to cover and some teaching tips. Preparation for anatomy also included how to handle students being upset by the dissection situation.)
University D, Sweden (*N*=4)		1st year 2nd year	4–12 4–7	Anatomy dissection Practical physiology labs		
University D, Sweden (*N*=3)		1st year 2nd year	4–12 4–7	Anatomy dissection Practical Physiology labs		
University D, Sweden (*N*=3)		1st year	4–12	Anatomy dissection		
University D, Sweden (*N*=2)		1st year 2nd year	4–12 4–7	Anatomy dissection Practical Physiology labs		

Since our aim in this article was twofold, we first present our findings related to peer teachers’ learning outputs followed by our findings related to the prerequisites for learning.

### Learning Outputs

We developed four themes concerning learning outputs: *Seeing teachers as facilitators of learning*, *Recognizing the limits of one’s own knowledge*, *Adapting teaching to learners needs*, and *Recognizing psychological safety as important for learning.*

#### Seeing Teachers as Facilitators of Learning

When new to teaching, peer teachers typically presumed they needed to be subject experts who could answer any question the peer learners had. Many expressed concerns about not being able to fulfil this role and they were worried about having insufficient subject knowledge. However, through pre-course preparation and/or teaching experience, their perception of their role shifted towards a more facilitative approach, focusing less on them taking center stage. Instead of acting as knowledge transmitters, they felt increasingly comfortable with helping peer learners do the work themselves, using strategies such as asking questions to stimulate reflection and initiating in-class discussions, as exemplified in the following quotes:

In the beginning I thought I would be more active. That I would kind of just stand and tell others what I knew […] it’s stressful that it would be like that. […] I’ve discovered later that it’s not how students learn best. (Participant 8A)

[The peer learners] often want you to just walk over to the cadaver and explain to them, from A to Z, all the muscles that are there and all the tendons and the arteries and everything. While I’m always trying to be like “Okay, can you maybe explain to me what you see?” […] Try to keep your cool and don’t just … spit out the answer the instant they ask you. (Participant 2B)

In addition to viewing facilitation as an efficient strategy to help students learn the course content, peer teachers were motivated by helping their peers to understand “…how to arrive at thinking, the actual thought process when you don’t know the answer right away” (Participant 9A). They stressed the importance of reasoning skills and wanted to help students develop their own strategies for analyzing problems.

#### Recognizing the Limits of One’s Own Knowledge

The teaching experience gave peer teachers insight into their own knowledge and its limitations. Working with peer learners forced them to revisit the course content which in turn reminded them of what they themselves once had learned. The discovery of how knowledge was retrievable was seen as a positive and motivating experience. At the same time, they were confronted with how much they had forgotten, for example, when trying to respond to detailed questions from peer learners. It was important for peer teachers not to misinform their peer learners. Hence, they expressed a moral obligation to critically scrutinize their own understanding before providing answers or giving advice:

I have sort of been able to identify more quickly: do I really know this or is it something I think I know? So, then you are transparent to the student all the time, and saying, ‘now I’m speculating’ and ‘now I go beyond what I know’. (Participant 7C)

I can do that in an exam, if I get a question that I don’t know the answer to I’ll just make something up in the hopes that it is correct. […] But you can’t do that when you teach a course, because then [the peer learners] think that it is correct. They trust you 100%! (Participant 5A)

These quotes illustrate how awareness of their own knowledge and its limitations led peer teachers to adopt a transparent approach when sharing knowledge with their peer learners. They reported being more comfortable with displaying knowledge gaps after engaging in peer teaching. These metacognitive reflections also enabled peer teachers to evaluate when they needed assistance from someone with greater expertise or when to seek guidance from the teaching staff.

#### Adapting Teaching to Learners’ Needs

Peer teachers developed ways to monitor their teaching effectiveness by assessing if their students in fact were learning. As part of this, they reported becoming increasingly aware of how their peer learners differed in academic level, teaching preferences, and learning needs. They also developed sensitivity to signals from learners such as facial expressions, body language, and how they responded. The peer teachers learned to use those signals as feedback.

To see that now they don’t understand what is going on. Even if they do not say anything, you see that they are no longer keeping up. (Participant 5A)

Other observations they learned to take advantage of were what types of questions the peer learners asked. These would give useful information about how much they understood.

I found out quickly that if I stand too long [and lecture] then it becomes … too much for them, and things I said at the beginning, they would ask about again. That means that they didn’t catch what I said, or that they forgot or did not understand. (Participant 2A)

Interestingly, the peer teachers did not necessarily view signals of learners struggling as negative or undesirable. They asserted that learning should or at least could involve some “resistance” and argued that the process of tumbling with difficult concepts and figuring things out themselves would lead to better learning.

Some peer teachers would adapt to peer learners by changing their teaching strategies or simplifying the course material. Others refrained from altering their teaching methods, arguing that even if students often expressed a preference for lecturing, they remained confident that student active and exploratory teaching would benefit their learners the most. Some peer teachers admitted struggling with adapting their teaching.

#### Recognizing Psychological Safety as Important for Learning

Peer teaching experiences helped students recognize how psychological safety is essential for learning. A feeling of safety was believed to facilitate peer learners’ active participation, as safety lessened their fear of failure. It was important for the peer teachers to build trust, and to be the person students could turn to. One of the participants elaborated on this:

It is nice to be the older student they know, because they might not know anyone else. And then you are the person they can … with whom it’s safe to ask: “How does it really work? What happens when you are in year five and about to write your student thesis? What is that?” (Participant 4C)

Two main approaches for establishing psychological safety were identified: One was to create safe and inclusive learning spaces and establish good social relationships with all students. This included making sure not to single students out by asking difficult questions in front of the class as this was believed to discourage students from speaking up.

I don’t like to expose anyone by asking a question [in plenary], like “What did I say now?”. (Participant 5C)

The second strategy was being a good role model. Peer teachers described how, in situations where students felt worried or overwhelmed by the demands of medical school, they could alleviate stress by personifying that it is possible to get through the hard work of medical school. By leveraging expectations and being transparent about their own knowledge gaps, they felt they could motivate students and reduce perfectionism.

### Prerequisites for the Learning Outputs

Four factors were found to be significant for the peer teachers’ learning: *Accessible and attractive teaching opportunities*, *Responsibility and agency*, *Peer teacher preparations*, and *Support during the course.*

#### Accessible and Attractive Teaching Opportunities

For students to reap the benefits of peer teaching they must get the opportunity to teach beyond occasional stand-alone situations. Our informants reported that despite being motivated to take on a job as a peer teacher, they struggled with finding time in their schedules to actually teach. Medical students have crowded schedules, especially in the final years, and it was hard for them to get exemption from obligatory courses and lectures. Some had quit teaching because of this.

I actually wanted to stay because I felt it was quite nice. The problem was that it became a bit of work at the very end of the year, and it was a bit like ... in the way of my own things. (Participant 3A)

I wish the faculty was a little more helpful and more understanding of the importance of peer teaching. (Participant 2A)

Busy schedules also hampered pre-course training and led peer teachers to opt for less lengthy and more time-efficient teacher training. Being paid for the job made peer teaching more attractive for the students.

#### Responsibility and Agency

The participants reported various degrees of micro-management or autonomy given to them as peer teachers. Peer teachers who described their roles as strictly predefined, with limited opportunities to make methodological or content-related decisions, reported gaining less learning from the experience. In contrast, peer teachers who experienced being trusted with responsibility to teach as they saw fit, tended more towards describing peer teaching as a learning experience. This entailed having to make decisions about teaching methods, what content to prioritize and sometimes administering the whole course including scheduling time, rooms, announcement, registration, and so forth.

Furthermore, peer teachers stressed that with agency comes responsibility. Many participants reported feeling that basic subject knowledge was a minimum requirement, while some also felt responsible for their peer learners’ well-being and learning outcomes.

The responsibility felt more like making sure everyone felt safe going there and catching those who were feeling bad. (Participant 9B)

Avoiding misinformation and providing help to find answers when they themselves fell short were considered important.

#### Peer Teacher Preparations

The amount and type of training offered to peer teachers as preparations varied greatly. Three different types of training were identified: subject-specific training, generic pedagogical training, and training to understand a specific peer teacher role. The most common preparatory intervention offered was subject-specific training for newly recruited peer teachers. This was found useful as it boosted their confidence in having the required basic knowledge needed to function as a teacher. When peer teachers were offered generic pedagogic training, this typically centered on student active teaching approaches. However, many missed more organized teacher training with focus on evidence-based teaching methods. In its absence, they admitted to using their previous teaching experiences (observing examples of good and bad faculty teachers), experiences as peer learners, or experience from their work in student organizations to guide their teaching.

Many informants wished that what was expected of them in the peer teacher role had been explicitly communicated, acknowledging that this would vary depending on what course they were teaching. Peer teachers who did receive role-specific pre-course training, catered to for instance being a PBL-facilitator, reported that they had a better understanding of their role, *why* they should use certain teaching methods, and seemed to grasp the idea that they were facilitators and not subject experts more quickly.

[After the peer teacher training] was when I understood what the role entailed. I think that if I hadn’t read about [PBL], I would have gone in with the mindset of answering questions and being the expert source. But then I realized that’s not the point. (Participant 4B)

#### Support During the Course

Support during the course refers to the mechanisms set in place to help peer teachers as their teaching unfolds. Peer teachers relied on a variety of resources for support when they had questions or experienced difficult situations, such as faculty members, more senior peer teachers, or same level peer teachers teaching the same course. Informants highlighted the value of co-teaching. Such collaborations seemed to build a sense of belonging to a team of peer teachers. Moreover, it allowed peer teachers to inspire each other as it facilitated discussions about teaching. They also appreciated and wanted more feedback from each other.

I would have thought it would be nice to […] as a first-time tutor, to go with someone who had been a tutor before. To run a physiology lab with someone who has already run that physiology lab so that you can wade into the role, I would have thought that would be the absolute best. (Participant 10C)

## Discussion

In this study, we aimed to investigate peer teaching experiences across a variety of contexts and programs to identify what are the generic learning outputs for the peer teachers. We found that peer teachers learned to view teaching differently, monitor teaching effectiveness, and adapt teaching to learners’ needs. Furthermore, they learned about the significance of psychological safety [19] in groups of learners and gained increased awareness of their own knowledge and skills. These findings provide nuance to previous research that has identified “increased pedagogical knowledge and skills” as a broad category of learning outputs for peer teachers [15]. More importantly, this is uplifting considering their future teaching roles as residents or faculty members. Additionally, pedagogical skills are likely to help students become more efficient and empathetic communicators in physician–patient interactions [20].

Our findings align with previous studies showing that peer teachers gain competencies corresponding to the CanMEDS competency framework, particularly the competencies of medical expert, collaborator, scholar, and professional [14]. Peer teachers develop their role as scholars by facilitating learning, adapting to peer learners, and fostering psychological safety. They also learn to be a professional by increasing their awareness of their own limitations and admitting to them [21]. Such metacognitive skills, recognized as the ability to plan, monitor, evaluate, and regulate one’s own learning processes [22], are important not only for learning efficiently through medical school, but also for the ability to continue developing key competencies in problem-solving, critical thinking, and self-directed learning throughout a medical career [23].

The prerequisites identified in our analysis are of particular interest as they bring attention to the factors associated with making peer teaching a learning experience for the peer teachers. We found that access to teaching opportunities, agency, teacher training, and attainable support are key factors relevant for the peer teaching learning experience. Teacher training has previously been found to improve peer teachers’ understanding of educational principles and to boost confidence in teaching [16]. Adding to this, our findings help distinguish between preparatory teacher training and continuous support while teaching. We argue that facilitating collaborations and discussions on teaching-related topics parallel to working as peer teachers cannot be replaced by preparatory teaching courses. Peer teachers seem to need both scaffolding mechanisms. Furthermore, our findings suggest that training and support should be non-directive as students appear to thrive with the responsibility they are entrusted with. Without the need to reflect and make decisions about how to teach, peer teachers learn less and are more prone to adopt a rote mindset. Challenge and reward appear to go hand in hand.

### Strengths and Limitations

Our study is strengthened by data sourced from different contexts, programs, subjects, and types of peer teaching, allowing us to identify commonalities and draw conclusions that extend beyond single case studies. However, we cannot claim that the peer teaching experience alone led to the described learning outputs, since these results may have been influenced by additional sources of learning. Recruitment bias is also likely to play a role, since most peer teachers are prone to be high-performing students. Hence, further studies are needed to test if all medical students will have the same or similar learning outputs from teaching their peers.

## Conclusion

In conclusion, our study adds to the growing evidence that being a peer teacher has the potential to grant beneficial learning outputs for medical students that will be useful in their future careers. These include how to facilitate learning in others and crucial insight into their own knowledge and its limitations. Our study also provides insight into the prerequisites that facilitate this learning process. Faculty members and course managers who intend to initiate or are already using students as teachers should pay particular attention to these factors in efforts to increase the value of peer teaching for all parties involved. For peer teaching to be successful, we recommend it to be not only encouraged, but also facilitated, supported, and reimbursed.

## Electronic supplementary material

Below is the link to the electronic supplementary material.

Supplementary file1 (DOCX 25 KB)

## Data Availability

The data that support the findings of this study are not publicly available due to privacy and confidentiality agreements with the informants. For further information, please contact the corresponding author.

## Appendix

### Interview Guide

What does the role as a peer teacher entail and how is it organized?

- Do you teach scheduled classes?

Tell us about the role as a peer teacher! What was it like to teach?

- What were your expectations of being a peer teacher?
- Was there anything that surprised you?
- Can you describe a situation where you felt you mastered or did not master the role of a peer teacher?

How did you teach the students?

- Can you describe a method that you felt worked well? Or worked poorly?
- Why did you choose your setup for teaching?
- Did you feel that the structure of the teaching worked well (reactions from students)?
- What do you think your students learned?
- Did you receive any feedback on your work? From whom and in what form?
- How was the cooperation with other peer teaches?

What have you learned from being a peer teacher?

- In what way has teaching influenced your professional development?
- How has peer teaching affected your own learning?
- In what way can your experiences be useful in your future profession as a doctor?
- Is there something you would have done differently if you were to teach again?

How would you describe cooperation with the course manager/teacher at the department?

- What type of assignment have you been given?
- How much responsibility did you get?
- How bound or free are you in your approach to teaching?
- Did you feel well taken care of by the course coordinator?

Did you attend any teacher training?

- If you received training, was it particularly useful?
- Have you missed any type of training for the teaching assignment you have had? (Both regarding subject knowledge and pedagogy). If yes, how do you think such education/training would best be designed?

Is there a question that was not included that you think I should have asked?

How did you experience this focus group interview?

Additional questions incorporated into the Swedish focus groups:

1. What do you think is your most important function as a peer teacher?
2. For whom is Peer-assisted learning best, the student or the teacher?
3. Is there a need for further training *after* starting an assignment? If so, how would it best be designed?
4. Are there other ways in which peer-learning could be used in the medical program?
5. Should it be mandatory for students in the medical program to teach other students/patients? Why/why not? If no; Should pedagogical training be compulsory in the medical program?
