# Supplementary material for: Learning Outputs for Peer Teachers in Undergraduate Medical Education
Source: Med Sci Educ. 2025 Mar 22;35(3):1617–26. doi: 10.1007/s40670-025-02365-0 (PMC12228852; doi:10.1007/s40670-025-02365-0)
Supplement: Supplementary file 1 — Supplementary file1 (DOCX 25 KB) [file 40670_2025_2365_MOESM1_ESM.docx]

**Appendix 1: Interview guide**

What does the role as a peer teacher entail and how is it organized?

- Do you teach scheduled classes?

Tell us about the role as a peer teacher! What was it like to teach?

- What were your expectations of being a peer teacher?
- Was there anything that surprised you?
- Can you describe a situation where you felt you mastered or did not master the role of a peer teacher?

How did you teach the students?

- Can you describe a method that you felt worked well? Or worked poorly?
- Why did you choose your setup for teaching?
- Did you feel that the structure of the teaching worked well (reactions from students)?
- What do you think your students learned?
- Did you receive any feedback on your work? From whom and in what form?
- How was the cooperation with other peer teaches?

What have you learned from being a peer teacher?

- In what way has teaching influenced your professional development?
- How has peer teaching affected your own learning?
- In what way can your experiences be useful in your future profession as a doctor?
- Is there something you would have done differently if you were to teach again?

How would you describe cooperation with the course manager/teacher at the department?

- What type of assignment have you been given?
- How much responsibility did you get?
- How bound or free are you in your approach to teaching?
- Did you feel well taken care of by the course coordinator?

Did you attend any teacher training?

- If you received training, was it particularly useful?
- Have you missed any type of training for the teaching assignment you have had? (Both regarding subject knowledge and pedagogy). If yes, how do you think such education/training would best be designed?

Is there a question that was not included that you think I should have asked?

How did you experience this focus group interview?

**Additional questions incorporated into the Swedish focus groups:**

1. What do you think is your most important function as a peer teacher?
2. For whom is Peer-assisted learning best, the student or the teacher?
3. Is there a need for further training *after* starting an assignment? If so, how would it best be designed?
4. Are there other ways in which peer-learning could be used in the medical program?
5. Should it be mandatory for students in the medical program to teach other students/patients? Why/why not? If no; Should pedagogical training be compulsory in the medical program?
